# Prescribing Patterns for Treatment of *Mycobacterium avium* Complex and *M. xenopi* Pulmonary Disease in Ontario, Canada, 2001–2013

**DOI:** 10.3201/eid2507.181817

**Published:** 2019-07

**Authors:** Sarah K. Brode, Hannah Chung, Michael A. Campitelli, Jeffrey C. Kwong, Alex Marchand-Austin, Kevin L. Winthrop, Frances B. Jamieson, Theodore K. Marras

**Affiliations:** West Park Healthcare Centre, Toronto, Ontario, Canada (S.K. Brode);; ICES, Toronto (S.K. Brode, H. Chung, M.A. Campitelli, J.C. Kwong);; University of Toronto, Toronto (S.K. Brode, J.C. Kwong, F.B. Jamieson, T.K. Marras);; University Health Network and Sinai Health System, Toronto (S.K. Brode, T.K. Marras);; Toronto Western Family Health Team, Toronto (J.C. Kwong);; Public Health Ontario, Toronto (J.C. Kwong, A. Marchand-Austin, F.B. Jamieson);; Oregon Health and Science University, Portland, Oregon, USA (K.L. Winthrop)

**Keywords:** Nontuberculous mycobacteria, Mycobacterium infections, nontuberculous, Mycobacterium avium complex, tuberculosis and other mycobacteria, treatment, Ontario, Canada, Mycobacterium xenopi, respiratory infections

## Abstract

Surveys suggest that clinicians diverge from guidelines when treating *Mycobacterium avium* complex (MAC) pulmonary disease (PD). To determine prescribing patterns, we conducted a cohort study of adults >66 years of age in Ontario, Canada, with MAC or *Mycobacterium xenopi* PD during 2001–2013. Using linked laboratory and health administrative databases, we studied the first treatment episode (>60 continuous days of >1 of a macrolide, ethambutol, rifamycin, fluoroquinolone, linezolid, inhaled amikacin, or, for *M. xenopi*, isoniazid). Treatment was prescribed for 24% MAC and 15% of *M. xenopi* PD patients. Most commonly prescribed was the recommended combination of macrolide, ethambutol, and rifamycin, for 47% of MAC and 36% of *M. xenopi* PD patients. Among MAC PD patients, 20% received macrolide monotherapy and 33% received regimens associated with emergent macrolide resistance. Although the most commonly prescribed regimen was guidelines-recommended, many regimens prescribed for MAC PD were associated with emergent macrolide resistance.

## Abstract 2

Although the most commonly prescribed treatment regimens follow guidelines, a substantial proportion of prescribed treatments foster macrolide resistance.

Nontuberculous mycobacteria (NTM) pulmonary disease (PD) is increasing in North America (*1*–*3*). The 2 most common causes of NTM PD in Ontario, Canada, are *Mycobacterium avium* complex (MAC) and *M. xenopi* (*1*). Treatment guidelines detailing evidence-based treatment regimens for MAC PD have been published; the first-line recommendation is a 3-drug combination of a macrolide, ethambutol, and a rifamycin (hereafter referred to as standard triple therapy) (*4*). Although there are no evidence-based treatment regimens for *M. xenopi* PD, expert-supported regimens have been suggested (*4*). Physician surveys suggest that, when treating MAC PD, clinicians frequently diverge from guideline recommendations (*5*,*6*). However, population-based data on treatment practices for MAC PD or *M. xenopi* PD are lacking. Our study objective was to examine antimicrobial drug prescribing patterns for MAC PD or *M. xenopi* PD in older Ontario residents.

## Methods

Our retrospective cohort study used population-based linked laboratory and health administrative databases in Ontario, Canada, described previously (*7*). These datasets were linked by using unique encoded identifiers and analyzed at ICES (Toronto, Ontario, Canada). Ontario is Canada’s most populous province; the population in 2013 was 13.5 million residents. Ontario has a single-payer healthcare system that provides universal access to medically necessary inpatient and outpatient services and prescription drugs to adults >65 years of age. Ontario also has a reference mycobacteriology laboratory that processes >95% of NTM specimens for the province (*8*).

Our study cohort consisted of adults >66 years of age with incident MAC PD or *M. xenopi* PD, defined according to American Thoracic Society/Infectious Diseases Society of America (ATS/IDSA) microbiological criteria (*4*), during 2001–2013; observations ended December 31, 2014. The date of diagnosis was defined as the date of collection of the first positive culture sample. To avoid confusion regarding the species for which the treatment was intended, we excluded patients who met ATS/IDSA microbiological criteria for infection with >1 NTM species during follow-up. We also excluded patients who died within 1 year of NTM PD diagnosis (and in a sensitivity analysis those who died within 2 years of diagnosis) and patients who had culture-confirmed tuberculosis (TB) after NTM PD diagnosis. We looked back 3 years before the study period to find preexisting isolation of NTM and *M. tuberculosis* complex; we excluded patients for whom NTM had been isolated during the 3-year look-back period and *M. tuberculosis* complex within 18 months of NTM PD diagnosis.

We studied the first treatment episode after NTM PD diagnosis, defined as >60 continuous days of treatment (either daily or intermittent) with >1 drug or class commonly used to treat MAC PD or *M. xenopi* PD (macrolide, ethambutol, rifamycin, fluoroquinolone, linezolid, inhaled amikacin, or, for *M. xenopi* PD, isoniazid), started within 1 year of any culture positive for the causative NTM species/complex and ended at the time of a >60 day treatment interruption. To allow for patients who refilled their prescriptions late, we defined treatment as continuous if they filled their next prescription for the same antimicrobial drug class within 1.5 times the number of days supplied in their last prescription. We also examined antimicrobial drug treatment given in the first 18 months after the start of the first treatment episode (i.e., not ending at a treatment interruption of >60 days) to capture breaks in therapy and switches between regimens (each defined as lasting >60 days).

We collected data about patient demographics and underlying conditions at the time of NTM PD diagnosis, prescribing physician specialty, treatment details, and medication use and healthcare use associated with asthma and chronic obstructive pulmonary disease (COPD) at the time of NTM PD diagnosis (Appendix) (*9*–*16*). To compare patient characteristics, we used analysis of variance for continuous variables and χ^2^ tests for categorical variables. To determine patient characteristics associated with initial prescription of macrolide monotherapy for >60 continuous days versus other regimens, we also performed bivariate and multivariable logistic regression analyses among MAC PD patients; included variables were selected a priori on the basis of clinical relevance. We used SAS version 9.4 (SAS Institute, https://www.sas.com) for all analyses and considered a 2-sided p value of <0.05 to be significant. This study was approved by research ethics boards at University Health Network and Public Health Ontario.

## Results

Of the 3,163 patients with MAC PD and 1,048 with *M. xenopi* PD, we excluded 329 (10.4%) MAC PD and 120 (11.4%) *M. xenopi* PD patients because they also met microbiological criteria for infection with another species of NTM PD or had TB. Treatment was received by 688 (24.2%) of the 2,834 patients with exclusively MAC PD and 142 (15.3%) of the 928 with exclusively *M. xenopi* PD. A sensitivity analysis limited to patients who survived >2 years after NTM PD diagnosis indicated that treatment was received by 622/2533 (24.6%) of MAC PD patients and 114/785 (14.5%) of *M. xenopi* PD patients. Compared with MAC PD patients who did not receive treatment, those who did receive treatment were younger (mean age 75.6 vs. 76.9 years); more likely to be female (59.4 vs. 54.8%); more likely to reside in neighborhoods in the higher income quintile and rural settings; more likely to have bronchiectasis, COPD, and interstitial lung disease; and less likely to have diabetes mellitus, chronic kidney disease, and lung cancer (Table 1). Compared with *M. xenopi* PD patients who did not receive treatment, those who did receive treatment were more likely to have COPD (83.1 vs 63.0%) (Table 1).

**Table 1:** Baseline characteristics of patients who did and did not receive treatment for MAC PD and *M. xenopi* PD, Ontario, Canada, 2001–2013*

Characteristic	MAC PD				*M. xenopi* PD		
	Treated, n = 688†	Untreated, n = 2,146	p value		Treated, n = 142†	Untreated, n = 786	p value
Sex			0.031				0.248
F	409 (59.4)	1,175 (54.8)			61 (43.0)	380 (48.1)	
M	279 (40.6)	971 (45.2)			81 (57.0)	407 (51.8)	
Age, mean ± SD	75.6 ± 5.94	76.9 ± 6.65	<0.001		75.1 ± 5.92	76.1 ± 6.50	0.077
Income quintile			0.018				0.932
1 (lowest)	142 (20.6)	550 (25.6)			32 (22.5)	164 (20.9)	
2	146 (21.2)	428 (19.9)			33 (23.2)	178 (22.6)	
3	124 (18.0)	425 (19.8)			26 (18.3)	131 (16.7)	
4	117 (17.0)	354 (16.5)			24 (16.9)	157 (20.0)	
5 (highest)	155–159 (22.5–23.1)	382 (17.8)			22–27 (15.5–19.01)	153–158 (19.5–20.1)	
Missing	<5 (<0.7)	7 (0.3)			<5 (<3.5)	<5 (<0.7)	
Residency‡			<0.001				0.288
Rural	37 (5.4)	53 (2.5)			≤5 (≤3.5)	8 (1.0)	
Suburban	89 (12.9)	159 (7.4)			9–14 (6.3–9.9)	38 (4.8)	
Urban	562 (81.7)	1,934 (90.1)			129 (90.8)	740 (94.1)	
ADGs, mean ± SD	10.4 ± 3.49	10.4 ± 3.67	0.775		11.3 ± 3.52	10.8 ± 3.77	0.117
Underlying conditions§							
Asthma	265 (38.5)	751 (35.0)	0.094		64 (45.1)	311 (39.6)	0.219
Bronchiectasis	169 (24.6)	335 (15.6)	<0.001		19 (13.4)	90 (11.5)	0.511
Chronic kidney disease	40 (5.8)	199 (9.3)	0.004		12 (8.5)	66 (8.4)	0.983
COPD	462 (67.2)	1,209 (56.3)	<0.001		118 (83.1)	474 (60.3)	<0.001
Cystic fibrosis	<5 (<0.7)	<5 (<0.2)	0.327		<5 (<3.5)	<5 (<0.6)	0.172
Diabetes mellitus	121 (17.6)	518 (24.1)	<0.001		28 (19.7)	206 (26.2)	0.101
GERD	139 (20.2)	429 (20.0)	0.903		31 (21.8)	158 (20.1)	0.638
HIV infection	<5 (≤0.7)	<5 (<0.2)	0.087		0	0	NA
Interstitial lung disease	81 (11.8)	138 (6.4)	<0.001		14 (9.9)	62 (7.9)	0.430
Lung cancer	19 (2.8)	135 (6.3)	<0.001		9 (6.3)	74 (9.4)	0.237
Prior TB	<5 (<0.7)	17 (0.8)	0.161		<5 (<3.5)	12 (1.5)	0.915
Rheumatoid arthritis	27 (3.9)	79 (3.7)	0.770		6 (4.2)	31 (3.9)	0.875

*Values are no. (%) except as indicated. According to privacy regulations, values representing <6 persons are reported as <5, and data are presented as a range of values for categorical variables where back-calculation is possible. ADGs, aggregated diagnostic groups (from the adjusted clinical group case mix system); COPD, chronic obstructive pulmonary disease; GERD, gastroesophageal reflux disease; MAC, *Mycobacterium avium* complex;, *M. xenopi*; *Mycobacterium xenopi*; NA, not applicable; PD, pulmonary disease; TB, tuberculosis. †Treated defined as >60 continuous days of treatment with >1 drugs/classes commonly used to treat MAC or *M. xenopi* PD (macrolide, ethambutol, rifamycin, fluoroquinolone, linezolid, inhaled amikacin, or, for *M. xenopi*, isoniazid), started within 1 y of any positive culture for the causative nontuberculous mycobacteria species/complex. ‡Derived from rural index for Ontario group, a measure of rurality designed for Ontario (*9*). §Defined according to inpatient and outpatient diagnostic codes in databases of hospital discharges and physicians’ services claims, respectively; see Appendix for definitions. Definitions have been validated for all underlying conditions with the exception of bronchiectasis and interstitial lung disease.

The median time from NTM PD diagnosis to start of the first treatment episode was 77 (interquartile range [IQR] 28–239) days for MAC PD and 79 (IQR 40–199) days for *M. xenopi* PD patients. Among MAC PD patients who received treatment, the most commonly prescribed drug in the first treatment episode was a macrolide (87.1%), followed by ethambutol (70.2%), a rifamycin (58.6%), and a fluoroquinolone (33.7%) (Table 2). These drugs were prescribed with similar frequency for *M. xenopi* PD patients. No linezolid was prescribed. Isoniazid, assessed for *M. xenopi* PD disease only, was rarely prescribed (<5 [<3.5%] patients). Amikacin, as recorded in our databases, was dispensed for inhalation and was rarely used; for <5 (<0.7%) MAC PD and 0 *M. xenopi* PD patients, inhaled amikacin for >60 days was prescribed.

**Table 2:** Proportion of patients with MAC PD and *M. xenopi* PD who had ever received each antimicrobial drug and select drug combinations and duration during first treatment episode, Ontario, Canada, 2001–2013*

Treatment†	MAC PD, n = 688			*M. xenopi* PD, n = 142	
	No. (%)	Mean duration ± SD, d		No. (%)	Mean duration ± SD, d
Individual drug					
Macrolide					
Any	599 (87.1)	447 ± 367		120 (84.5)	359 ± 312
Clarithromycin	318 (46.2)	369 ± 364		66 (46.5)	319 ± 367
Azithromycin	354 (51.5)	424 ± 348		66 (46.5)	335 ± 244
Rifamycin					
Any	403 (58.6)	437 ± 366		69 (48.6)	349 ± 228
Rifampin	384 (55.8)	434 ± 363		61 (43.0)	351 ± 233
Rifabutin	30 (4.4)	323 ± 285		9 (6.3)	294 ± 217
Ethambutol	483 (70.2)	456 ± 357		84 (59.2)	363 ± 284
Fluoroquinolone					
Any	232 (33.7)	369 ± 353		63 (44.4)	312 ± 189
Moxifloxacin	82 (11.9)	318 ± 382		27 (19.0)	251 ± 195
Levofloxacin	56 (8.1)	226 ± 239		11 (7.7)	240 ± 282
Ciprofloxacin	137 (19.9)	328 ± 333		36 (25.4)	283 ± 181
Gatifloxacin	≤5 (≤0.7)	126 ± 163		0	NA
Norfloxacin	8 (1.2)	197 ± 288		0	NA
Linezolid	0	NA		0	NA
Isoniazid	NA	NA		<5 (<3.5)	160 ± 110
Drug regimen					
Standard triple: macrolide + ethambutol + rifamycin ± others	326 (47.4)	369 ±269		51 (35.9)	241 ± 173
Macrolide + ethambutol	91 (13.2)	315 ± 283		11 (7.7)	159 ± 83
Macrolide + rifamycin	49 (7.1)	284 ± 392		10 (7.0)	251 ± 208
Macrolide + fluoroquinolone	65 (9.4)	267 ± 278		20 (14.1)	228 ± 153
Other macrolide-containing combinations‡	115 (16.7)	346 ±276		31 (21.8)	295 ± 156
Nonmacrolide combination§	63 (9.2)	258 ± 298		19 (13.4)	198 ± 187
Macrolide monotherapy	141 (20.5)	262 ± 358		34 (23.9)	330 ± 509
Nonmacrolide monotherapy	201 (29.2)	206 ± 226		40 (28.2)	253 ± 207
No. drugs given, mean ± SD	2.5 ± 0.9			2.4 ± 1.0	
No. drugs given					
1	142 (20.6)			37 (26.1)	
2	121 (17.6)			24 (16.9)	
3	372 (54.1)			69 (48.6)	
4	53 (7.7)			12 (8.5)	
No. switched regimens¶					
0	401 (58.3)			85 (59.9)	
1	177 (25.7)			33 (23.2)	
2	72 (10.5)			14 (9.9)	
>3	38 (5.5)			10 (7.0)	
Maximum no. drugs used at any 1 time, mean ± SD	2.4 ± 0.9			2.3 ± 0.9	

*MAC, *Mycobacterium avium* complex; M*. xenopi*, *Mycobacterium xenopi*; PD, pulmonary disease. †>60 continuous d of treatment with >1 drugs/classes commonly used to treat MAC PD or *M. xenopi* PD (macrolide, ethambutol, rifamycin, fluoroquinolone, linezolid, inhaled amikacin, or for *M. xenopi* PD, isoniazid), started within 1 y of any positive culture for the causative nontuberculous mycobacteria species/complex. Patients may have received >1 drug from an antibiotic class, and/or>1antibiotic regimen during their first treatment episode, so values do not add up to 100%. According to privacy regulations, values representing <6 persons are reported as <5, and data are presented as a range of values for categorical variables where back-calculation is possible. ‡>2 drugs excluding: macrolide + fluoroquinolone, macrolide + EMB, macrolide + rifamycin. §>2 drugs (e.g., ethambutol, a rifamycin, a fluoroquinolone (or for *M. xenopi* PD, isoniazid), without macrolide. ¶Change in treatment lasting >60 d, in the first treatment episode.

We examined the proportion of patients for whom particular regimens had ever been prescribed for >60 days during their first treatment episode. Among patients with treated MAC PD, the guidelines-recommended standard triple regimen (macrolide/ethambutol/rifamycin) was the most commonly prescribed (47.4%), followed by nonmacrolide monotherapy (29.2%) and macrolide monotherapy (20.5%) (Table 2). Drug regimens associated with development of macrolide-resistant MAC (macrolide monotherapy, macrolide/fluoroquinolone, and macrolide/rifamycin) (*17*,*18*) were prescribed for 224/688 (32.6%) of MAC PD patients. Among *M. xenopi* PD patients who received treatment, standard triple therapy was prescribed for 35.9%, followed by nonmacrolide monotherapy for 28.2%, and macrolide monotherapy for 23.9% (Table 2).

The flow of antimicrobial drug treatment during the first 18 months (i.e., regimen sequence, duration, and transitions) revealed that, among MAC PD patients who received treatment, the most common starting regimen was standard triple therapy (290/688; 42.1%) (Figure), prescribed for a mean (± SD) of 315 (± 167) days (median 334; IQR 151–467 days) before a regimen switch or discontinuation. Among MAC PD patients for whom the initial regimen was associated with development of macrolide resistance, these regimens were prescribed for the following mean (± SD) durations before a switch or discontinuation: macrolide monotherapy, 230 (± 167) days; macrolide/fluoroquinolone, 216 (± 147) days; and macrolide/rifamycin, 197 (± 139) days (Figure). For a large minority of MAC PD patients, therapy was switched during the first treatment episode; >1 regimen was switched for 41.7% (Table 2; Figure). Among MAC PD patients who received treatment, 50.2% received treatment for >12 months before discontinuation. Among *M. xenopi* PD patients who received treatment, 31.0% initially received standard triple therapy and 40.1% underwent ≥1 regimen switch (Table 2).

**Figure:**
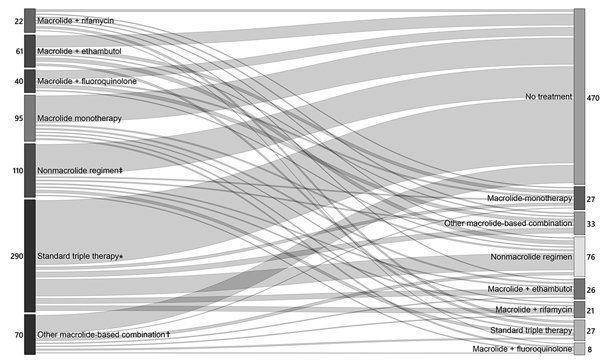
Flow of therapy for 688 patients with *Mycobacterium avium* complex pulmonary disease, depicting transition between first and second regimens during first 18 months of treatment, Ontario, Canada, 2001–2013. Values are the number of patients receiving each treatment regimen in each epoch of therapy. An epoch is defined as >60 days of the therapy. The width of the lines is proportional to the number of patients receiving and transitioning between each regimen. Mean (± SD) duration of treatment, in days, for each starting regimen is as follows: standard triple therapy 315 (± 167), other macrolide containing combination 331 (± 157), macrolide-ethambutol 274 (± 172), macrolide monotherapy 230 (±167), nonmacrolide containing regimen 176 (± 178), macrolide-fluoroquinolone 216 (± 147), macrolide-rifamycin 197 (± 139). *Macrolide, ethambutol, and a rifamycin, ± other drugs; †macrolide + >2 additional drugs (other than standard triple therapy); ‡ethambutol, a rifamycin, or fluoroquinolone, either alone or in combination.

Among MAC PD patients who received treatment, for their first regimen, the specialties of the main prescribing physicians varied. The prescriber was a pulmonologist for 55.7%, an infectious diseases specialist for 10.0%, an internal medicine specialist for 7.4%, a family physician/general practitioner for 12.3%, and another specialist or of unknown specialty for 14.5% (Table 3).

**Table 3:** Initial treatment regimen, by prescriber specialty, for 688 patients with *Mycobacterium avium* complex pulmonary disease, Ontario, Canada, 2001–2013*

Regimen	Specialty, no. (%) patients				
	Respirology, n = 383 (55.7)	ID, n = 69 (10.0)	GIM, n = 51 (7.4)	FP/GP, n = 85 (12.3)	Other/unknown, n = 100 (14.5)
Standard triple therapy	166 (43.3)	37 (53.6)	22 (43.1)	34 (40.0)	31 (31.0)
Macrolide monotherapy	55 (14.4)	7 (10.1)	<5 (≤9.8)	14 (16.5)	15 (15.0)
Macrolide + rifamycin or fluoroquinolone	38 (9.9)	≤5 (≤7.2)	≤5 (<9.8)	6 (7.1)	8 (8.0)
Other	124 (32.4)	20–25 (29.0–36.1)	20 (39.2)	31 (36.5)	46 (46.0)

*Includes the regimen dispensed for at least the first 60 d of treatment. According to privacy regulations, values representing<6 persons are reported as <5, and data are presented as a range of values for categorical variables where back-calculation is possible. GIM, general internal medicine; FP/GP, family practice/general practice; ID, infectious diseases.

According to bivariate analyses, patients with MAC PD whose initial regimen was macrolide monotherapy were more likely than those whose initial regimen was anything else to have asthma or COPD , to have received a long-acting bronchodilator or oral corticosteroid in the prior year, to have visited an emergency department or been hospitalized in the prior 2 years for an asthma or COPD exacerbation, to have received oxygen at home, and to have received pulmonary function tests in the previous 5 years (Table 4). However, according to adjusted analyses, only use of oral corticosteroids in the prior year was significantly associated with a starting regimen of macrolide monotherapy (adjusted odds ratio 2.01, 95% CI 1.16–3.50).

**Table 4:** Characteristics of MAC PD patients according to initial treatment regimen, Ontario, Canada, 2001–2013*

Characteristic	Macrolide monotherapy, n = 95†	Other regimen, n = 593	Unadjusted OR (95% CI)	p value	Adjusted OR (95% CI)‡	p value
Sex						
F	52 (54.7)	357 (60.2)	0.80 (0.52–1.24)	0.314	0.92 (0.57–1.48)	0.738
M	43 (45.3)	236 (39.8)	Referent	NA	Referent	NA
Age, mean ± SD	76.21 ± 6.67	75.52 ± 5.81	1.02 (0.98–1.06)	0.292	1.03 (0.99–1.07)	0.123
Income quintile						
1 (lowest)	19 (20.0)	123 (20.7)	Referent	0.682	NA	NA
2	22 (23.2)	124 (20.9)	1.15 (0.59–2.23)	0.470	NA	NA
3	13 (13.7)	111 (18.7)	0.76 (0.36–1.61)	0.945	NA	NA
4	16 (16.8)	101 (17.0)	1.03 (0.50–2.10)	0.752	NA	NA
5 (highest)	21–25 (22.1–26.3)	134 (22.6)	1.11 (0.58–2.14)	0.986	NA	NA
Missing data	<5 (≤2.1)	0	NA		NA	NA
Residency§						
Rural	<5 (<2.1)	35 (5.9)	0.33 (0.08–1.40)	0.132	NA	NA
Suburban	8–12 (8.4–12.6)	79 (13.3)	0.73 (0.36–1.47)	0.378	NA	NA
Urban	83 (87.4)	479 (80.8)	Referent	NA	NA	NA
ADGs, mean ± SD	10.45 ± 3.90	10.36 ± 3.42	1.01 (0.95–1.07)	0.805	NA	NA
Underlying conditions¶						
Asthma	47 (49.5)	218 (36.8)	1.68 (1.09–2.60)	0.019	1.22 (0.72–2.07)	0.451
Bronchiectasis	27 (28.4)	142 (23.9)	1.26 (0.78–2.05)	0.347	1.19 (0.71–1.99)	0.504
Chronic kidney disease	7 (7.4)	133 (5.6)	1.35 (0.58–3.15)	0.486	NA	NA
COPD	75 (78.9)	387 (65.3)	2.00 (1.19–3.36)	0.009	1.47 (0.81–2.66)	0.208
Diabetes mellitus	21 (22.1)	100 (16.9)	1.40 (0.82–2.38)	0.214	NA	NA
GERD	21 (22.1)	118 (19.9)	1.14 (0.68–1.93)	0.619	NA	NA
Interstitial lung disease	14 (14.7)	67 (11.3)	1.36 (0.73–2.53)	0.335	NA	NA
Lung cancer	<5 (<2.1)	17 (2.9)	0.73 (0.17–3.21)	0.676	NA	NA
Rheumatoid arthritis	<5 (<2.1)	25 (4.2)	0.49 (0.11–2.10)	0.335	NA	NA
Drug exposure within 1 y#						
Short-acting BD	49 (51.6)	271 (45.7)	1.27 (0.82–1.95)	0.287	0.69 (0.38–1.26)	0.225
Long-acting BD	52 (54.7)	248 (41.8)	1.68 (1.09–2.60)	0.019	1.16 (0.56–2.39)	0.694
ICS	55 (57.9)	287 (48.4)	1.47 (0.95–2.27)	0.087	0.89 (0.42–1.87)	0.754
OCS	38 (40.0)	130 (21.9)	2.37 (1.51–3.74)	<0.001	2.01 (1.16–3.50)	0.013
Methylxanthine	9 (9.5)	28 (4.7)	2.11 (0.96–4.63)	0.062	1.52 (0.64–3.57)	0.340
ED visit/hospitalization for asthma or COPD within 2 y#	22 (23.2)	88 (14.8)	1.73 (1.02–2.93)	0.042	0.92 (0.48–1.77)	0.799
Prior/current home oxygen therapy	12 (12.6)	31 (5.2)	2.62 (1.30–5.31)	0.007	1.83 (0.84–3.98)	0.128
PFTs within 5 y#	78 (82.1)	416 (70.2)	1.95 (1.12–3.39)	0.018	1.52 (0.82–2.78)	0.180
Pulmonologist prescriber	55 (57.9)	328 (55.3)	1.11 (0.72–1.72)	0.638	1.03 (0.66–1.63)	0.889

*Values are no. (%) except as indicated. According to privacy regulations, values representing <6 persons are reported as <5, and data are presented as a range of values for categorical variables where back-calculation is possible. ADGs, aggregated diagnostic groups (from the adjusted clinical group case mix system); BD, bronchodilator; COPD, chronic obstructive pulmonary disease; ED, emergency department; GERD, gastroesophageal reflux disease; ICS, inhaled corticosteroid; MAC, *Mycobacterium avium* complex; NA, not applicable; NTM, nontuberculous mycobacteria; OCS, oral corticosteroid; OR, odds ratio; PD, pulmonary disease; PFTs, pulmonary function tests. †Macrolide monotherapy was the first antibiotic regimen given after NTM PD diagnosis, and was considered ≥60 d with no companion drugs of interest. ‡Variables were selected for inclusion in the multivariable model a priori, based on clinical relevance. §Derived from rural index for Ontario group, a measure of rurality designed for Ontario (*9*). ¶Defined according to inpatient and outpatient diagnostic codes in databases of hospital discharges and physicians’ services claims, respectively. Definitions have been validated for all underlying conditions with the exception of bronchiectasis and interstitial lung disease. #Before NTM PD diagnosis.

## Discussion

In this population-based study of treatment practices for MAC PD and *M. xenopi* PD in adults >66 years of age, we found that a minority of patients received antimicrobial therapy: 24% of MAC PD patients and 15% of *M. xenopi* PD patients. During the first treatment episode, the most commonly prescribed regimen, initially and overall, was standard triple therapy. However, it is concerning that many MAC PD patients received >60 days of treatment with regimens associated with macrolide resistance, a situation that is extremely difficult to treat and associated with high mortality rates (*17*–*19*). Macrolide monotherapy was prescribed for 20% of MAC PD patients, and other regimens associated with facilitating macrolide resistance (macrolide/fluoroquinolone, macrolide/rifamycin) were also frequently prescribed. Although standard triple therapy was prescribed initially for 42% and ever (during the first treatment episode) for 47% of patients, regimens that facilitate macrolide resistance were prescribed initially for 23% and ever for 33%. Treatment flow was complex, and switches between regimens were common.

In our study, the proportion of patients with MAC PD who received antimicrobial drug treatment (24%) was lower than that described by others. Studies from South Korea (*20*) and Germany (*21*) reported treatment rates within 3 years of diagnosis of 65% for MAC PD and 74% for NTM PD patients. In Oregon, USA, treatment was initiated within 2 years of diagnosis for 54% of NTM PD patients (*22*). According to physician survey studies, antimicrobial drug treatment was received by 55% of MAC PD patients in the United States (*5*), 68% of NTM PD patients from 5 countries in the European Union (*6*), and 43% of NTM PD patients in Japan (*6*). The reasons why a relatively small proportion of MAC PD patients in our study received treatment are probably many. First, our definition of NTM PD (based on microbiological criteria only) probably more often misclassified patients from whom NTM were repeatedly isolated as having disease, compared with the Oregon study, which reviewed all diagnostic criteria (*22*), and the Germany study, which used diagnostic codes (*21*). Second, our study was population based and thereby included the full spectrum of disease severity and physician expertise, compared with specialty clinic-based studies (*20*), which probably comprise patients with more severe disease and physicians who may be more likely to treat NTM PD because of greater experience. Third, we included only adults >66 years of age; older patients may be less likely to receive treatment, as was noted among the MAC PD patients in this study and has been described by others (*20*). Some patients may not have been prescribed treatment because of a limited life expectancy resulting from underlying conditions; however, when we performed a sensitivity analysis limited to patients who survived >2 years after NTM PD diagnosis, we found no notable change in the proportion who received treatment. Fourth, we required 1 positive culture for the causative NTM within 1 year of treatment initiation and >60 continuous days of dispensed prescriptions, which were not requirements for the other studies. Of note, the proportion of patients who received treatment in our study was similar to the 18% of NTM PD patients who received treatment at 4 integrated US healthcare delivery systems (*23*), in which patients were identified by using a combination of culture results and codes from the International Classification of Diseases, Ninth Revision.

We found associations between baseline characteristics and receipt of MAC PD treatment. Income distribution was significantly associated with treatment; patients residing in neighborhoods in lower income quintiles seemed less likely to receive treatment. Although Canada provides universal access to medically necessary health services, including prescription drugs for adults >65 years of age, socioeconomic disparities in access to specialist care have been observed (*24*) and may play a role. We also found that patients living in urban settings were less likely to receive treatment; this finding is somewhat surprising in that others have shown that urban patients are more likely than rural patients to receive ambulatory care, including specialist care, for other chronic medical conditions (*25*,*26*). Whether a disparity in the proportion of patients with true disease exists when comparing patients with MAC isolates from urban versus rural settings is not clear.

We found prescription of standard triple therapy for MAC PD to be more common (47% ever received it and 42% received it as initial therapy) than that reported in the United States (13% ever received) (*5*), the European Union (9% for >6 months) (*6*), and Germany (19%) (*21*) but similar to that reported in Japan (42% for >6 months) (*6*). The possible reasons for these differences include differences in study methods, prescribing physician specialty, financial coverage for medications, and familiarity with ATS/IDSA guidelines. Regarding physician specialties, the proportion of MAC PD patients receiving treatment from a pulmonologist in our study (57%) was similar to that in Japan (54%) (*6*) but higher than that in the European Union (29%) (*6*) and in the United States (37%) (*5*). Pulmonologists may be more aware of the ATS/IDSA guidelines than are other specialists. However, pulmonologists in Ontario (43%) seemed more likely than those in the United States (18%) to prescribe standard triple therapy for MAC PD (*5*), which may result from different patient populations and medication coverage. We included only adults >66 years of age because this population has comprehensive medication coverage. Pulmonologists in the United States may prescribe nonstandard antimicrobial drug regimens for patients who do not have prescription drug coverage because of cost.

In our study, 20% of MAC PD patients who received treatment were prescribed >60 days of macrolide monotherapy, 9% >60 days of macrolide/fluoroquinolone, and 7% >60 days of macrolide/rifamycin therapy. Findings of studies of physicians in the United States (*5*) and Germany (*21*) were similar. These regimens are associated with development of macrolide resistance (*17*,*18*); resistance developed in 20% of 59 patients who received macrolide monotherapy for 4 months compared with 4% of 303 patients who received standard triple therapy (*17*). In our study, among 95 MAC PD patients whose initial regimen was macrolide monotherapy, the mean duration was 230 days; macrolide/fluoroquinolone and macrolide/rifamycin regimens were given for similar durations. Therefore, the regimen duration was long enough to constitute a risk for macrolide resistance.

For some patients, these drugs may have been prescribed for other conditions. This possibility applies especially to macrolide monotherapy, which may have been prescribed to treat exacerbations of asthma, COPD, or bronchiectasis and may not have been prescribed to treat MAC per se. This possibility is supported by our analyses; bivariate analysis indicated that presence of asthma and COPD were associated with receipt of macrolide monotherapy versus another regimen. Although bronchiectasis was not associated with receipt of macrolide monotherapy, our databases contain no validated bronchiectasis definition, and the definition we used (1 physician billing claim or hospitalization with bronchiectasis diagnosis) seems to be of very low sensitivity, given the small number of patients with NTM assigned a code for bronchiectasis. Also, some treatments for asthma/COPD (long-acting bronchodilators, oral corticosteroids, home oxygen), as well as emergency department visits/hospitalizations for asthma/COPD, were associated with prescription of macrolide monotherapy. According to multivariable analyses, the only variable associated with prescription of macrolide monotherapy was receipt of oral corticosteroids <1 year before MAC PD diagnosis, which is consistent with the possibility that macrolide monotherapy was prescribed to prevent asthma/COPD exacerbations. It is possible that some patients for whom macrolide monotherapy was prescribed for asthma, COPD, or bronchiectasis did not have clinical or radiologic findings of MAC PD. However, these patients did fulfill microbiological criteria for MAC PD and had >1 positive culture within 1 year before filling the prescription. No data describe the risk of inducing macrolide resistance in persons with positive sputum cultures who do not meet full diagnostic criteria for MAC PD. However, the fact that one fifth of patients in our study who received treatment and met microbiological criteria for MAC PD received this regimen for >60 days is concerning. Given the increasing use of macrolides for asthma, COPD, and bronchiectasis, further research into the safety of these drugs in patients with NTM isolation is needed.

Few data exist regarding prescribing patterns for patients with *M. xenopi* PD. A retrospective study of 136 patients in France who had *M. xenopi* PD meeting full ATS/IDSA diagnostic criteria found that 59% received treatment (*27*) compared with 15% in our study. That study found that patients’ initial treatment regimens contained an average of 4 drugs among rifamycins (88%), ethambutol (75%), isoniazid (66%), clarithromycin (30%), and fluoroquinolones (21%). In our study, patients received fewer drugs (mean 2.4 ± 1.0 SD) and were more likely to receive a macrolide (84%) or a fluoroquinolone (44%) and less likely to receive a rifamycin (49%), ethambutol (59%), or isoniazid (<4%). The difference between prescribing patterns for *M. xenopi* PD in France versus Ontario may be partially explained by the periods of the studies (1983–2003 in France vs. 2001–2013 in Ontario); evidence supporting the efficacy of macrolides for treating *M. xenopi* infection emerged in the mid-1990s (*28*–*31*). Also, the study in France was not restricted to older adults and included only patients at 1 of 13 hospitals, which may have limited them to more severe cases. Another possible explanation relates to differences in the more geographically proximal treatment guidelines; the 1999 British Thoracic Society guidelines recommended treatment with rifampin and ethambutol ± isoniazid (*32*), whereas the 1997 and 2007 ATS/IDSA guidelines recommend a regimen of clarithromycin, rifampin, and ethambutol (*4*,*28*), ± isoniazid ± moxifloxacin (*4*).

For MAC PD and *M. xenopi* PD patients who received treatment, regimen switches were common (42% of MAC PD patients underwent >1 regimen switch). Others have reported similar findings; in 2 case series of MAC PD patients who received standard triple therapy, regimens were switched for 46%–71% of patients receiving daily therapy and 3%–21% of patients receiving intermittent therapy (*33*,*34*). We did not study daily versus intermittent therapy and are not able to draw conclusions regarding tolerability of different drugs or combinations. However, our finding of frequent regimen switches suggests that drug intolerance was common and may partially explain the frequent use of regimens not recommended in treatment guidelines.

Our study has several limitations. We based our definition of NTM PD on microbiological criteria alone and therefore probably misclassified some patients as having true disease, possibly contributing to the observation that a low proportion of patients received treatment. We defined treatment as >60 days of an antimicrobial drug of interest being dispensed; this definition may not capture some patients in whom there was an intent to treat, such as patients who had medications prescribed but never dispensed and patients who received treatment for NTM PD but stopped taking the medication in <60 days. Because we included only adults >66 years of age, our findings may not apply to younger patients. Last, we were not able to study the use of clofazimine or injectable aminoglycosides because the relevant information is not contained in our databases. This omission may have caused us to erroneously label some patients as having received an inappropriate regimen when the regimen was strengthened by clofazimine or an aminoglycoside. However, because clofazimine is not approved for use in Canada and was difficult to access during the study period, we think that these patients are probably few. 

The use of inhaled amikacin seemed to be rare. We also excluded patients who met diagnostic criteria for NTM PD associated with >1 species. The proportion was small, combined with exclusions for TB amounting to 10.4% for MAC PD and 11.1% for *M. xenopi* PD. Given the species distribution of NTM in Ontario (MAC PD and *M. xenopi* PD comprising the overwhelming majority of treated NTM episodes), the very high similarity between MAC PD and *M. xenopi* PD treatments, and our lack of data for intravenous treatments needed to analyze *M. abscessus* therapy, we elected to exclude these patients.

In summary, the most commonly prescribed regimen for MAC PD and *M. xenopi* PD in Ontario was standard triple therapy. This finding is somewhat reassuring; however, a large minority of patients with MAC PD received regimens that may lead to macrolide resistance. Physicians who treat patients with NTM PD should take care to follow established treatment guidelines for management of this condition.

Appendix Definitions of underlying conditions used in study of prescribing patterns for treatment of *Mycobacterium avium* complex and *M. xenopi* pulmonary disease, Ontario, Canada, 2001–2013.
