# Supplementary material for: Prescribing Patterns for Treatment of Mycobacterium avium Complex and M. xenopi Pulmonary Disease in Ontario, Canada, 2001–2013
Source: Emerg Infect Dis. 2019 Jul;25(7):1271–80. doi: 10.3201/eid2507.181817 (PMC6590764; doi:10.3201/eid2507.181817)
Supplement: Appendix — Definitions of underlying conditions used in study of prescribing patterns for treatment of Mycobacterium avium complex and M. xenopi pulmonary disease, Ontario, Canada, 2001–2013. [file 18-1817-Techapp-s1.pdf]

Article DOI: https://doi.org/10.3201/eid2507.181417

Prescribing Patterns for Treatment of *Mycobacterium avium* Complex and *M. xenopi* Pulmonary Disease in Ontario, Canada, 2001–2013

Appendix

Definitions used for each underlying condition

- Asthma: ≥2 physician billing claims and/or ≥1 hospital discharges with a diagnosis of asthma within 2 years, in accordance with the following codes: 493 (OHIP and International Classification of Diseases, Ninth Revision [ICD-9] codes) or J45 (ICD-10-CA codes) from April 1, 1991, to the index date (*1*)

- Bronchiectasis: ≥1 physician billing claim and/or ≥1 hospital discharges with a diagnosis of bronchiectasis in accordance with the following codes: 494 (OHIP and ICD-9 codes) or J47, Q33.4, Q89.3 (ICD-10-CA codes) from April 1, 1991, to the index date

- Chronic kidney disease:

- Patients with a diagnosis of CKD (*2*) within 5 years before index date, using the following diagnostic codes: 403, 585 (OHIP); 4030, 4031, 4039, 4040, 4041, 4059, 585, 586, 5888, 5889, 2504 (ICD-9); E102, E112, E132, E142, I12, I13, N08, N18, N19 (ICD-10)

OR

- Patients who were on chronic dialysis (*3*) from April 1, 1991 to index date, identified as those with at least 2 of any of the following codes in OHIP, CIHI-DAD, or CIHI-SDS separated by at least 90 days, but less than 150 days

OHIP service codes: R849, G323, G325, G326, G860, G862, G865 G863, G866, G330, G331, G332, G333, G861, G082, G083, G085, G090, G091, G092, G093, G094, G095, G096, G294, G295, G864, H540, H740

CIHI-DAD, CIHI-SDS: CCI procedure codes: 5195, 6698, CCP procedure code: 1PZ21

- COPD: ≥1 physician billing claim and/or ≥1 hospital discharges with a diagnosis of COPD in accordance with the following codes: 491, 492, or 496 (OHIP and ICD-9 codes) or J41, J43, or J44 (ICD-10-CA codes) from April 1, 1991, to the index date (*4*).

- Cystic fibrosis: ≥1 hospitalization with either of ICD-9 diagnosis of 277 or ICD-10 diagnosis of E84.X from April 1, 1991, to the index date

- Diabetes mellitus: ≥2 physician billing claims or ≥1 OHIP service code or ≥1 hospital discharges with a diagnosis of diabetes within 2 years, in accordance with the following codes: 250 (OHIP and ICD-9 codes), Q040, K029, K030, K045, K046 (OHIP service codes), or E10, E11, E13, E14 (ICD-10-CA codes) from April 1, 1991, to the index date (*5*)

- GERD: ≥1 hospitalization with either ICD-9 diagnosis of 530.1 or 530.8 or ICD-10 diagnosis of K21.0 or ≥1 physician billing claim with OHIP code 530, from Apr 1, 1991 to the index date (*6*)

- HIV: ≥3 physician billing claims with a diagnosis of HIV within 3 years, in accordance with the following OHIP/ICD-9 codes: 042, 043, or 044, from April 1, 1991, to the index date (*7*)

- Interstitial lung disease: ≥1 physician billing claim and/or >1 hospital discharges with a diagnosis of interstitial lung disease in accordance with the following codes: 515 (OHIP and ICD-9 codes) or J84.0-J84.9 (ICD-10-CA codes) from April 1, 1991, to the index date.

- Lung cancer: identified using the Ontario Cancer Registry, where the diagnosis date was before index date (OCR topography code ‘C34’).

- Prior TB: ≥1 isolate culture positive for *M. tuberculosis* complex between January 1, 1998 and index date

- Rheumatoid arthritis: ≥3 physician billing claims with ≥1 by a musculoskeletal specialist (rheumatologist, orthopedic surgeon, or general internist) within 3 years, or ≥1 hospital discharges with a diagnosis of rheumatoid arthritis, in accordance with the following codes: 714 (OHIP/ICD-9) or M05, M06 (ICD-10-CA codes) from April 1, 1991, to the index date (*8,9*)

- Definition of Rurality/Urbanity: The Rurality Index of Ontario (RIO) score, version 2008, is a continuous measure of rurality designed for use in Ontario which includes a combination of population size and density, travel time to referral centers, availability of various medical services, general metropolitan facilities and infrastructure, and climatic extremes (*10*). This index was used to classify patients as residing in urban (RIO 2008 score 0–9), suburban (RIO 2008 score 10–39), or rural (RIO 2008 score 40+) communities.

References

1. Gershon AS, Wang C, Guan J, Vasilevska-Ristovska J, Cicutto L, To T. Identifying patients with physician-diagnosed asthma in health administrative databases. Can Respir J. 2009;16:183–8. PubMed http://dx.doi.org/10.1155/2009/963098

2. Fleet JL, Dixon SN, Shariff SZ, Quinn RR, Nash DM, Harel Z, et al. Detecting chronic kidney disease in population-based administrative databases using an algorithm of hospital encounter and physician claim codes. BMC Nephrol. 2013;14:81. PubMed http://dx.doi.org/10.1186/1471-2369-14-81

3. Quinn RR, Laupacis A, Austin PC, Hux JE, Garg AX, Hemmelgarn BR, et al. Using administrative datasets to study outcomes in dialysis patients: a validation study. Med Care. 2010;48:745–50. PubMed http://dx.doi.org/10.1097/MLR.0b013e3181e419fd

4. Gershon AS, Wang C, Guan J, Vasilevska-Ristovska J, Cicutto L, To T. Identifying individuals with physcian diagnosed COPD in health administrative databases. COPD. 2009;6:388–94. PubMed http://dx.doi.org/10.1080/15412550903140865

5. Hux JE, Ivis F, Flintoft V, Bica A. Diabetes in Ontario: determination of prevalence and incidence using a validated administrative data algorithm. Diabetes Care. 2002;25:512–6. PubMed http://dx.doi.org/10.2337/diacare.25.3.512

6. Lopushinsky SR, Covarrubia KA, Rabeneck L, Austin PC, Urbach DR. Accuracy of administrative health data for the diagnosis of upper gastrointestinal diseases. Surg Endosc. 2007;21:1733–7. PubMed http://dx.doi.org/10.1007/s00464-006-9136-1

7. Antoniou T, Zagorski B, Loutfy MR, Strike C, Glazier RH. Validation of case-finding algorithms derived from administrative data for identifying adults living with human immunodeficiency virus infection. PLoS One. 2011;6:e21748. PubMed http://dx.doi.org/10.1371/journal.pone.0021748

8. Widdifield J, Bernatsky S, Paterson JM, Tu K, Ng R, Thorne JC, et al. Accuracy of Canadian health administrative databases in identifying patients with rheumatoid arthritis: a validation study using the medical records of rheumatologists. Arthritis Care Res (Hoboken). 2013;65:1582–91. PubMed

9. Widdifield J, Bombardier C, Bernatsky S, Paterson JM, Green D, Young J, et al. An administrative data validation study of the accuracy of algorithms for identifying rheumatoid arthritis: the influence of the reference standard on algorithm performance. BMC Musculoskelet Disord. 2014;15:216. PubMed http://dx.doi.org/10.1186/1471-2474-15-216

10. Kralj B. Measuring “rurality” for purposes of health-care planning: an empirical measure for Ontario. Ontario Medical Review. 2000;10:33–52.
